# PIWIL3/OIP5-AS1/miR-367-3p/CEBPA feedback loop regulates the biological behavior of glioma cells: Erratum

**DOI:** 10.7150/thno.69398

**Published:** 2022-01-21

**Authors:** Xiaobai Liu, Jian Zheng, Yixue Xue, Hai Yu, Wei Gong, Ping Wang, Zhen Li, Yunhui Liu

**Affiliations:** 1 Department of Neurosurgery, Shengjing Hospital of China Medical University, Shenyang, 110004, People's Republic of China;; 2 Liaoning Clinical Medical Research Center in Nervous System Disease, Shenyang, 110004, People's Republic of China;; 3 Key Laboratory of Neuro-oncology in Liaoning Province, Shenyang, 110004, People's Republic of China;; 4 Department of Neurobiology, College of Basic Medicine, China Medical University, Shenyang, 110122, People's Republic of China;; 5 Key Laboratory of Cell Biology, Ministry of Public Health of China, China Medical University, Shenyang, 110122, People's Republic of China;; 6 Key Laboratory of Medical Cell Biology, Ministry of Education of China, China Medical University, Shenyang, 110122, People's Republic of China.

The authors regret that the original version of our paper unfortunately contained some incorrect representative images. The transwell images in Figure 2J and Figure 4G, and the GAPDH protein band of U251 cells in Figure 3K had been misused during figure assembly. The correct version of the Figure 2J, Figure 3K and Figure 4G appears below.

The authors confirm that the corrections made in this erratum do not affect the original conclusions. The authors apologize for any inconvenience that the errors may have caused.

## Figures and Tables

**Figure 2:**
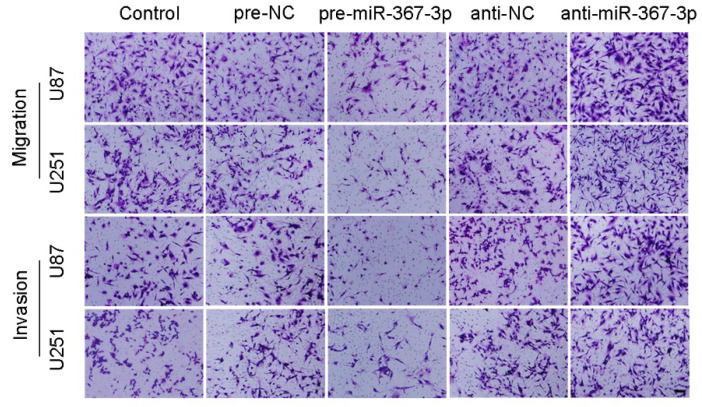
(J) Quantification of number of migrating and invading cells with over-expression or knockdown of miR-367-3p. Representative images and corresponding statistical plots are presented (scale bars represent 80 μm).

**Figure 3:**
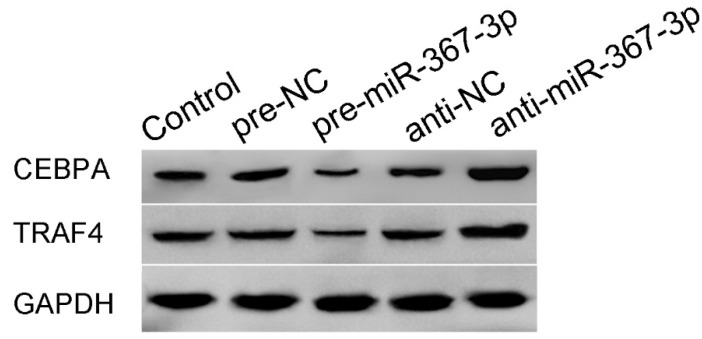
(K) Western blot analysis for miR-367-3p-regulated CEBPA expression in U251 cells. The relative expression of CEBPA is shown using GAPDH as an endogenous control.

**Figure 4:**
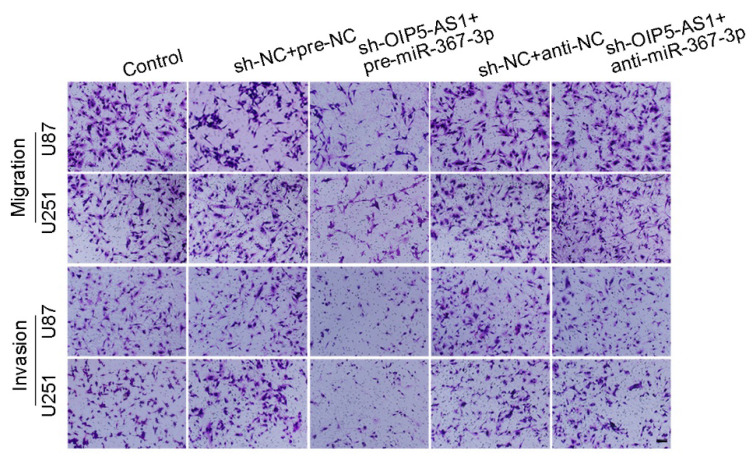
(G) Quantification of number of migrating and invading cells with altered expression of OIP5-AS1 and miR-367-3p. Representative images and corresponding statistical plots are presented (scale bars represent 80μm).

